# Exploring the interplay between host genetics and acute and long COVID: A narrative review

**DOI:** 10.1016/j.clinsp.2025.100708

**Published:** 2025-06-20

**Authors:** Thais Beuren, Filipe Ferrari, Leandro Tolfo Franzoni, Cássia da Luz Goulart, Fernando Val, Gerson Cipriano, Ricardo Stein

**Affiliations:** a Graduate Program in Cardiology and Cardiovascular Sciences, School of Medicine, Universidade Federal do Rio Grande do Sul, Porto Alegre, RS, Brazil; b Physiological Science Department, Universidade Federal do Amazonas, Manaus, AM, Brazil; c Health Sciences and Technologies Graduate Program, Universidade de Brasília (UnB), Brasília, DF, Brazil; d Graduate Program in Tropical Medicine, Universidade do Estado do Amazonas, Manaus, AM, Brazil; e Graduate Program in Human Movement and Rehabilitation of Evangelical (PPGMHR), UniEVANGÉLICA, Anápolis, GO, Brazil; f Department of Internal Medicine, Universidade Federal do Rio Grande do Sul, Porto Alegre, RS, Brazil

**Keywords:** Genetic predisposition, COVID-19, SARS-CoV-2, Pulmonary immunity

## Abstract

Over the past four years, pivotal discoveries have deepened the understanding of the relationship between genetic factors and SARS-CoV-2 infection. Numerous genes associated with severe COVID-19 suggest a potential genetic predisposition, which may help explain why some individuals develop more serious illnesses. Emerging evidence highlights the role of genes involved in pulmonary immunity, such as Forkhead box Protein P4 (FOXP4), whose increased expression in lung tissue has been linked to more severe disease. Other genes – Transmembrane Protease Serine-2 (TMPRSS2), Leucine Zipper Transcription Factor Like-1 (LZTFL1), Solute Carrier family 6 member 20 (SLC6A20), Tyrosine Kinase-2 (TYK2), Angiotensin-Converting Enzyme (ACE), and FYVE and Coiled-Coil Domain-Containing-1 (FYCO1) – have also been implicated in COVID-19 severity. In contrast, certain genetic variants – such as the T-allele of rs12329760 in the TMPRSS2 gene and rs35705950-T in the Mucin-5B (MUC5B) gene – may confer protection against severe disease. Overall, the evidence suggests that genetic factors can influence both susceptibility to and protection from severe COVID-19, although these associations are likely shaped by complex interactions with environmental, behavioral, and other biological factors. This review summarizes current knowledge on genetic determinants linked to COVID-19 outcomes.

## Highlights

• Genetic variants may affect individual COVID-19 severity.
• Some polymorphisms worsen outcomes; others may offer protection.
• Gene effects likely interact with environmental and biological factors.

## Introduction

Recent advancements in identifying genetic factors associated with severe COVID-19 and susceptibility to SARS-CoV-2 infection – particularly through Genome-Wide Association Studies (GWAS) – have been pivotal in enhancing the understanding of disease mechanisms and guiding the development of personalized treatment strategies.

Genetic factors have emerged as key determinants of the clinical spectrum of COVID-19, influencing not only susceptibility to infection and disease severity but also potential protective response in certain individuals. Specific genes, including critical regulators of pulmonary immunity – such as Forkhead box Protein-P4 (FOXP4) – have been implicated in shaping individual responses to the virus.^1^^,^^2^ GWAS have provided valuable insights into the complex genetic architecture of COVID-19.^3^ In addition to FOXP4, other genes – such as Transmembrane Protease Serine-2 (TMPRSS2), Leucine Zipper Transcription Factor-Like-1 (LZTFL1), Solute Carrier family-6 member-20 (SLC6A20), Tyrosine Kinase-2 (TYK2), and FYVE and coiled-coil domain-containing 1 (FYCO1) – have been associated with COVID-19 outcomes, contributing to both increased risk and potential resilience.^4^, ^5^, ^6^

Genetic predisposition has thus emerged as a central determinant of individual susceptibility to COVID-19. However, most reviews on the topic have focused on a limited number of genes and polymorphisms. In this narrative review, we examine recent findings on genetic factors associated with COVID-19, including variants linked to both increased severity and protective effects. We also recognize that these associations are likely shaped by multifactorial interactions involving environmental, behavioral, and biological influences.

To support this narrative review, we conducted a literature search in PubMed for English-language articles addressing genes and polymorphisms associated with COVID-19, published up to April 20, 2024. The search terms included: ‘COVID-19′ or ‘SARS-CoV-2′ and ‘genetic’ or ‘genetic factor’ or ‘gene’ or ‘polymorphism’ or ‘allele’. Reference lists of the included studies were also screened to identify additional potentially eligible articles. Observational studies, longitudinal studies, and meta-analyses were considered for inclusion. Given the narrative nature of this review, the use of a structured protocol and adherence to the Preferred Reporting Items for Systematic Reviews and Meta-Analyses (PRISMA) guidelines were not applicable.

## Genes associated with COVID-19 susceptibility or severity

### FOXP4

The FOXP4 gene is expressed in several tissues throughout the body, including the lungs, heart, brain, liver, and kidneys.^7^ It has been identified as an important regulator of the immune response in the lungs.^8^ Additionally, it plays a pivotal role in the regenerative capacity of lung epithelial cells, influencing mucus production and providing protection against pathogens.^9^

Current evidence indicates a direct correlation between high levels of FOXP4 expression in lung tissue and disease severity in COVID-19. D’Antonio et al.^10^ investigated interactions between COVID-19 phenotypes, genetic variations, and gene expression across 48 human tissues and 21 blood cell types. Among their key findings, the variant rs1886814, located 10 kb upstream of the FOXP4 gene, exhibited the highest probability of causality. Furthermore, in a cohort comprising 13,641 cases and over 2 million controls, FOXP4-AS1 was identified as a locus significantly associated with COVID-19 hospitalization.^2^

In a more recent study, Zhang et al.^11^ investigated 314 individuals with COVID-19 in Guangdong Province, China. Participants were divided into two groups: those with severe or critical disease (‘cases’; *n* = 64) and those with mild or moderate disease (‘controls’; *n* = 250). The study found that the rs1886814 and rs2894439 variants of the FOXP4 gene were associated with a significantly higher risk of severe COVID-19 (OR = 3.75; 95 % CI 1.7 to 8.0; *p* = 0.001 and OR = 5.7; 95 % CI 2.0 to 15.9; *p* = 0.001, respectively).

Studies have also explored genetic risk factors associated with long COVID, characterized by persistent symptoms following recovery from SARS-CoV-2 infection. A GWAS involving 6450 patients from 16 countries identified a significant association between the FOXP4 gene locus and the development of long COVID.^12^

Finally, FOXP4 plays a dual role in pulmonary biology – regulating lung epithelial regeneration and modulating immune responses. Its expression is particularly elevated in type 2 alveolar epithelial cells, which are essential for surfactant production and act as key mediators of the lung’s innate immune defense.^13^ In severe COVID-19, aberrant or prolonged FOXP4 expression may impair epithelial repair, sustain inflammation, or promote fibrotic remodeling.^13^ FOXP4 is also expressed in immune cells, including T-lymphocytes, and contributes to adaptive immune responses, particularly memory T-cell function. Although its role in the differentiation of Th1, Th2, and Th17 subsets is not yet fully understood, FOXP4 may influence immune modulation through T-cell-mediated mechanisms.^14^

Thus, the association between FOXP4 expression and COVID-19 severity may reflect a pathophysiological feedback loop, wherein impaired epithelial regeneration and immune dysregulation mutually exacerbate lung injury.^10^^,^^11^ Further mechanistic studies – such as those using conditional knockout models – are warranted to elucidate the causal pathways and assess the therapeutic relevance of FOXP4 in COVID-19 pathogenesis.

### OAS1

The 2′−5′-Oligoadenylate Synthetase-1 (OAS1) gene encodes an interferon-stimulated enzyme that plays a central role in antiviral defense. Upon recognizing viral double-stranded RNA (dsRNA), OAS1 is activated and catalyzes the conversion of ATP into 2′−5′-linked oligoadenylates (2–5A). These signaling molecules bind to and activate RNase L, a latent endoribonuclease that degrades viral and host single-stranded RNA, thereby inhibiting viral protein synthesis and promoting apoptosis in infected cells.^15^^,^^16^ Certain OAS1 splice variants, such as p46, exhibit enhanced antiviral activity against coronaviruses due to their localization to intracellular membranes, facilitating access to viral replication sites.^17^ Genetic polymorphisms affecting OAS1 expression or splicing – such as rs10774671 – have been associated with differences in susceptibility to and severity of COVID-19.^17^ Additionally, the OAS region has been identified as a locus associated with increased susceptibility to COVID-19 severity.^18^

Recent studies have illuminated the role of genetic variants within the OAS1 gene in modulating COVID-19 severity, particularly among individuals of European and African descent. A large-scale investigation involving 2249 individuals of European and 835 of African ancestry revealed an association between specific OAS1 variants and disease severity.^19^ Individuals hospitalized for COVID-19 were more likely to carry particular combinations of these variants compared to those with milder disease who did not require hospitalization. The study identified a common haplotype associated with reduced OAS1 expression, which correlated with greater COVID-19 severity. Furthermore, a shared haplotype containing human-specific risk alleles from two OAS1 variants was also linked to an increased risk of hospitalization.^19^

Similar findings were reported in a Slovak study involving 139 hospitalized COVID-19 patients and 63 healthy individuals of European origin.^20^ Additionally, specific OAS1 variants – such as rs10735079, rs6489867, and rs4767027 – have been implicated in susceptibility to SARS-CoV-2 infection, further underscoring the complex relationship between genetic factors and COVID-19 outcomes.^21^

While these studies provide valuable insights, most have focused on individuals of European and African ancestry. It remains unclear whether these findings are generalizable to other populations, such as those of Asian or Latin American descent, where distinct genetic architectures may influence the role of OAS1 variants in COVID-19 susceptibility and severity. Expanding research to include underrepresented populations is essential to address this gap and to develop a more comprehensive understanding of the global impact of OAS1 variations.

Moreover, it is important to recognize that the OAS1 gene region contains both risk-enhancing and protective variants. Some have been associated with reduced OAS1 expression and worse clinical outcomes, while others may confer resilience to infection or mitigate disease severity. These findings highlight the complex and sometimes opposing roles of OAS1 variants in modulating host responses to viral infection, underscoring the need for a nuanced interpretation of genetic associations.

In summary, current evidence underscores the significance of genetic variation within the OAS1 gene in influencing susceptibility to and severity of COVID-19.

### IFNAR2

Interferon, a key protein in the body’s innate response to viral threats, plays a vital role in host defense. Numerous studies have investigated the role of the Interferon Alpha and Beta Receptor subunit-2 (IFNAR2) gene in the context of COVID-19.

IFNAR2 has been implicated in disease severity,^22^ with Mendelian randomization studies suggesting a potential causal relationship between genetic variations in IFNAR2 and COVID-19 severity.^23^^,^^24^ Proposed mechanisms involve impaired innate antiviral defenses, particularly in the early stages of infection, linking IFNAR dysfunction to critical illness.^23^

A study by Zhang et al.^25^ identified genetic defects – including mutations in IFNAR2 – in at least 3.5 % of patients with life-threatening COVID-19 pneumonia. Loss-of-function mutations in IFNAR2 were associated with increased susceptibility to severe infection, as demonstrated through whole-genome sequencing analyses of samples from hospitalized patients requiring supplemental oxygen.^26^ Additionally, IFNAR2 protein concentrations were found to be elevated in patients with severe COVID-19 during the early phase of symptoms, further supporting its role in disease progression.^27^

Polymorphisms in IFNAR2, such as rs2236757, rs2834158, rs3153, and rs1051393, have also been linked to an increased risk of COVID-19 mortality.^28^ However, while loss-of-function mutations are associated with greater severity, elevated IFNAR2 levels have likewise been observed in severe cases, presenting a paradox that remains unresolved in current studies. This apparent inconsistency warrants further investigation.

A study conducted in Palestine investigated associations between IFNAR2 polymorphisms and both susceptibility to SARS-CoV-2 infection and disease severity. Participants were categorized into three groups: a control group (RT-PCR negative; *n* = 52), a community case group (RT-PCR positive; *n* = 70), and a severe case group (intensive care unit group; *n* = 32). Genotyping was performed using next-generation sequencing. Although the results suggested an association between the rs2236757A allele and increased risk of severe manifestations,^29^ the study’s small sample size (154 participants) and lack of discussion regarding potential sources of bias limit the generalizability of the findings.

Similarly, the same allele (rs2236757A) was associated with adverse outcomes in a retrospective study of 694 patients in Porto Alegre, Brazil (414 critical, requiring intensive unit care, and 280 non-critical). The association was more pronounced among women and non-white individuals.^30^

Additional insights have revealed differential expression of IFNAR2 and related genes in circulating leukocytes from SARS-CoV-2-infected individuals. These genes were upregulated in lymphocytes and monocytes from COVID-19 patients compared to healthy controls, indicating their involvement in the immune response.^31^ Notably, IFNAR2 variants demonstrated monocyte-specific, COVID-19-related quantitative expression trait effects, highlighting the influence of host genetic variation on immune cell behavior and disease severity.^32^

Taken together, accumulating evidence underscores the pivotal role of INAR2 in determining susceptibility to and severity of COVID-19. Collectively, these findings suggest that IFNAR2 concentrations in COVID-19 patients may serve as predictive indicators of disease progression.

### TYK2

Tyrosine Kinase-2 (TYK2) plays a crucial role in regulating immune responses.^33^ Variants in the TYK2 gene have been identified as potential risk factors for critical illness and hospitalization due to COVID-19.^2^ Supporting this, one study reported that carriers of the TYK2 rs74956615 variant exhibit increased genetic susceptibility to severe COVID-19, which is strongly associated with elevated levels of Intercellular Adhesion Molecule-1 (ICAM-1).^34^

Increased ICAM-1 levels have been observed in both plasma and post-mortem lung tissues of COVID-19 patients. A study by Zabihi Rizi et al.^35^ extracted genomic DNA from 200 individuals infected with SARS-CoV-2, dividing them into severe (*n* = 100) and mild (*n* = 100) disease groups. Their findings suggest that TYK2 single nucleotide polymorphisms may serve as genetic markers for identifying individuals at increased risk of severe COVID-19. Specifically, the rs2304255 T-allele was significantly associated with greater disease severity (OR = 3.24), as was the rs12720354 A allele (OR = 3.97). Based on these results, the authors proposed a hypothesis to explain the biological mechanisms linking TYK2 polymorphisms to disease severity.

However, it is important to note that this study^35^ did not account for potential confounding variables known to influence COVID-19 outcomes, such as age, comorbidities, and environmental factors. This lack of adjustment for these confounders limits the generalizability of the findings. Moreover, although the study establishes an association between TYK2 polymorphisms and disease severity, it does not fully explore the molecular mechanisms by which TYK2 variations alter immune signaling or affect disease progression. Further research into the functional role of TYK2 in immune responses to SARS-CoV-2 could provide valuable insights into the mechanistic pathways underlying this association.

In summary, the identification of TYK2 gene variants as predictors of COVID-19 severity has important implications for risk stratification and the development of targeted therapeutic interventions.

### TMPRSS2

TMPRSS2, a serine protease regulated by androgens, cleaves the spike protein of SARS-CoV-2. Evidence suggests that viral entry into the lung cells – a critical step for infection and replication – relies on interaction with TMPRSS2.^36^^,^^37^ In murine models infected with SARS-CoV, TMPRSS2 has been shown to play a pivotal role in viral spread within the airways.^38^

A meta-analysis identified a significant association between the TMPRSS2 rs12329760 variant and increased risk of severe COVID-19.^39^ Similarly, a study by Yaghoobi et al.^40^ demonstrated a strong link between the minor T-allele of rs12329760 and higher susceptibility to severe COVID-19 among Iranian patients. These findings were corroborated by another independent study in the same sample,^41^ further reinforcing the role of TMPRSS2 genetic variants in disease severity within specific backgrounds.

In Brazil, a genetic study involving over 400 hospitalized COVID-19 patients provided additional insights into the influence of TMPRSS2 variants on disease severity and mortality.^42^ Interestingly, older individuals carrying the rs2070788 GG genotype exhibited a four-fold higher risk of death compared to those with AG or AA genotypes. Multivariable analysis confirmed the study-independent association between the GG genotype and mortality, highlighting the prognostic value of TMPRSS2 polymorphisms in predicting clinical outcomes. However, in contrast to previous findings, no significant association was observed between the rs12329760 polymorphism and mortality in this Brazilian cohort.^42^

The conflicting associations of the rs12329760 variant in TMPRSS2 (a missense substitution, V160M) with COVID-19 severity across populations may reflect a complex interplay of genetic, hormonal, and virological factors. First, the variant may be in linkage disequilibrium with different functional alleles depending on ancestry, resulting in divergent biological effects.^40^ Second, TMPRSS2 is regulated by Androgen Receptor (AR) signaling, and variations in sex hormone levels, AR pathway genes, or epigenetic regulation may modulate the variant’s impact on gene expression.^43^^,^^44^ These interactions could explain why rs12329760 appears protective in high-androgen contexts but deleterious in others. Third, the V160M substitution may affect the protease’s efficiency in cleaving the SARS-CoV-2 Spike protein – a process potentially influenced by viral strain differences or the predominance of alternative entry pathways, such as cathepsin-mediated endocytosis.^45^^,^^46^ Methodological differences across studies, including population structure, sample size, and clinical definitions, may further contribute to these discrepancies. Collectively, these observations underscore the need for stratified analyses and functional studies to clarify how ancestry-specific genetic architecture and hormonal milieu shape the phenotypic effects of TMPRSS2 variants in COVID-19.

Although the Brazilian study highlights the prognostic relevance of TMPRSS2 variants, it does not explore the molecular mechanisms through which these polymorphisms influence TMPRSS2 function in the context of SARS-CoV-2 infection. Understanding how specific variants affect viral entry and subsequent immune responses could offer important insights into COVID-19 pathophysiology and enhance the predictive utility of genetic markers.

In conclusion, genetic studies and meta-analyses have identified a compelling association between TMPRSS2 variants and COVID-19 severity, although further research is needed to elucidate population-specific effects and the underlying biological mechanisms.

### LZTFL1

LZTFL1, a gene expressed in lung epithelial cells and located in chromosomal region 3p21.3, plays a vital role in regulating the epithelial-mesenchymal transition, a key response pathway to viral infection.^47^

Researchers have applied a combination of machine learning and molecular biology platforms to analyze GWAS, uncovering a connection between LZTFL1 and adverse outcomes in COVID-19.^48^ In a study conducted in Colombia involving 145 patients aged 18 to 60, the LZTFL1 rs11385942 polymorphism was identified as a significant risk factor for hospitalization (OR = 5.7; 95 % CI 1.2 to 27.0).^5^ In contrast, a study by Rüter et al.^49^ found a stronger association with the rs73064425 polymorphism in the LZTFL1 gene, linking the T-allele to an increased risk of infection and greater susceptibility to SARS-CoV-2. However, it is important to note that these two polymorphisms – rs11385942 and rs73064425 – are not directly comparable, as they are related to different aspects of the disease (risk of hospitalization vs. susceptibility to infection). The functional relationship between these variants, if any, remains unclear and warrants further investigation to determine whether they act independently or through a shared molecular mechanism.

Moreover, while some studies highlight risk alleles such as the T-allele in rs73064425, others emphasize differences within the LZTFL1 gene. This raises the possibility that LZTFL1 may influence disease outcomes through multiple distinct biological pathways. The identification of different risk alleles across studies remains partially unexplained and may reflect population-specific genetic backgrounds, environmental influences, or methodological differences in study design. These variables should be carefully considered when interpreting the findings and assessing the broader implications of LZTFL1 genetic variants in COVID-19 pathogenesis.

A study by Udomsinprasert et al.^50^ further explored the potential role of LZTFL1 variants in the manifestation of long-term symptoms following SARS-CoV-2 infection. Associations were observed between the rs10490770, rs11385942, and rs17713054 variants and the development of long COVID. Notably, specific alleles were linked to increased susceptibility to prolonged symptoms, underscoring the multifaceted impact of LZTFL1 variation on COVID-19 outcomes.

Collectively, these findings on LZTFL1 gene variants represent an important step forward in understanding the genetic determinants of COVID-19. By elucidating the role of LZTFL1 in both acute disease severity and long-term sequelae, current evidence provides valuable insights that may inform future research and guide clinical decision-making.

### SLC6A20

Located on chromosome 3p21.3 in humans, the SLC6A20 gene belongs to the SLC6 family of membrane transporters and contributes to the synthesis of integral transporter proteins involved in the sub-amino acid transporter system.^51^

Research has established a connection between SLC6A20 and both the risk and severity of COVID-19.^52^^,^^53^ In a genome-wide CRISPR loss-of-function study conducted by Kasela et al.,^52^ SLC6A20 emerged as a pivotal gene influencing COVID-19 severity. Using CRISPR technology to systematically knock out genes across the genome, the study identified SLC6A20 as a key factor associated with disease progression. However, details regarding the experimental conditions and the rationale for prioritizing SLC6A20 over other candidates were not thoroughly discussed in the original publication. Greater methodological transparency would help contextualize the role of SLC6A20 within the broader network of genes implicated in COVID-19 pathogenesis.

Positioned within a genetic cluster that includes other functionally relevant genes, SLC6A20 is believed to exert a significant influence on disease progression, suggesting its potential as a therapeutic target for mitigating severe COVID-19 outcomes.^53^

A meta-analysis further confirmed a significant association between polymorphisms in SLC6A20 and LZTFL1 and an increased risk of severe outcomes from viral infections (OR = 1.8; 95 % CI 1.5 to 2.0).^54^ Specific SLC6A20 variants – such as rs73062389-A and rs2271616-T – were associated with increased infection risk (OR = 1.2; 95 % CI 1.1 to 1.3 and OR = 1.15; 95 % CI 1.1 to 1.17, respectively).^54^

The identification of SLC6A20 as a key determinant of COVID-19 severity carries important implications for disease management and the development of targeted therapies. In summary, the current body of evidence supports a significant role for SLC6A20 in influencing the clinical course of COVID-19.

### FYCO1

FYCO1 demonstrates a specific protein – protein interaction with SARS-CoV-2, offering mechanistic insights into its role in viral pathogenesis. Transcriptomic data support the hypothesis that FYCO1 facilitates viral intracellular replication by associating with double-membrane vesicles derived from the endoplasmic reticulum – the primary site of SARS-CoV-2 replication.^55^ Additionally, FYCO1 is involved in interactions with the microtubule network, underscoring its multifaceted role in viral replication and intracellular trafficking.^56^

Compelling evidence supports FYCO1 as a central player in the pathogenesis of SARS-CoV-2. In a study by Gusakova et al.,^55^ the tertiary structure of FYCO1 was analyzed in a large, representative sample of the Russian population. The findings revealed a genetic predisposition to severe COVID-19 associated with FYCO1 variants. These results suggest that FYCO1 may promote viral replication and contribute to excessive exocytosis, thereby amplifying the risk of severe clinical outcomes.

Corroborating this, another study identified a potential causal relationship between the expression of several genes – including FYCO1 in specific immune cell types – and susceptibility to or severity of COVID-19. This conclusion was supported by transcriptome-wide association studies and colocalization analyses.^57^ It was proposed that FYCO1 expression in immune cells may influence both disease severity and susceptibility to SARS-CoV-2 infection.

Further supporting its role in viral susceptibility, Azzarà et al.^58^ identified FYCO1 as a gene associated with increased vulnerability to SARS-CoV-2. In their study, the number of FYCO1 variants present in each individual correlated with the degree of susceptibility to infection.

In summary, FYCO1 has been recognized as a key genetic mediator of heightened susceptibility and adverse outcomes in COVID-19. Through an integrative approach involving protein-protein interactions, GWAS, and transcriptomic analyses, researchers have begun to unravel the complex relationship between FYCO1 expression and disease progression, offering valuable insights into the genetic determinants of COVID-19 severity.

### MBL2

The Mannose-Binding Lectin-2 (MBL2) gene, located on chromosome 10, encodes the Mannose-Binding Lectin (MBL) glycoprotein, a key component of the innate immune response. Polymorphisms in the MBL2 gene have been implicated as contributing factors to susceptibility to infectious diseases.^59^^,^^60^

In a study conducted in Brazil, Queiroz et al.^61^ identified a significant association between polymorphisms in exon-1 of MBL2 and COVID-19 severity. Individuals with the homozygous polymorphic genotype (OO) exhibited a higher frequency of severe symptoms compared to those with milder presentations (15 % vs. 4 %, respectively; *p* = 0.02). Moreover, carriers of the OO genotype had an increased likelihood of developing severe disease, with an OR of 3.9 (95 % CI 1.34 to 11.28). However, the small sample size (*n* = 100 children) may limit the statistical power and generalizability of these findings. While the association observed was significant, caution is warranted, and replication in larger cohorts is necessary to validate these results.

Similar trends were observed in a Japanese cohort, where individuals carrying the BB genotype faced a heightened risk of mortality from COVID-19 compared to those with AA or AB genotypes (OR = 5.7; 95 % CI 1.10 to 29.05).^62^ This finding further underscores the potential prognostic value of MBL2 gene polymorphisms in predicting disease severity and mortality across distinct populations.

Polymorphisms in MBL2 have also been examined in pediatric populations. Yilmaz et al.^59^ reported a significant correlation between the codon 54 polymorphism in exon-1 and symptomatic COVID-19 among 100 children (mean age: 11-years). The AB and BB genotypes were more frequent in symptomatic patients (45.6 % and 23.5 %, respectively) than in asymptomatic individuals (9.4 % and 6.3 %, respectively; *p* < 0.001). Furthermore, the B-allele was significantly more prevalent in symptomatic cases (46.3 %) than in asymptomatic ones (10.9 %; *p* < 0.001).

These findings were corroborated by another study examining the codon 54 A/B (Gly54Asp; rs1800450) polymorphism in exon-1, which included 284 COVID-19 patients and 100 controls.^63^ The BB genotype was significantly more common among COVID-19 cases than controls (10.9 % vs. 1.0 %; *p* = 0.001). Compared to individuals with the AA genotype (used as the reference), those with BB or AB genotypes had a greater risk of severe disease and were more likely to require intensive care unit admission. In addition, the B-allele (rs1800450) – associated with MBL deficiency – was linked to a higher risk of pneumonia and hospitalization.^64^ These results further emphasize the prognostic significance of MBL2 polymorphisms in COVID-19 outcomes.

Variability in findings across different studies may be influenced by differences in genotype distribution (e.g., OO, BB), which are likely affected by population-specific genetic heterogeneity, ethnic background, environmental exposures, and circulating viral variants. Such factors may account for inconsistencies in the observed associations and should be carefully considered when interpreting results. Understanding these contextual influences is essential for elucidating how genetic variation in MBL2 impacts disease susceptibility, immune response, and clinical outcomes.

### ACE

The angiotensin-converting enzymes ACE1 and ACE2 are homologous but functionally distinct components of the renin-angiotensin-aldosterone system, each playing a different role in the pathophysiology of COVID-19. ACE1 converts angiotensin I into angiotensin II, a vasoconstrictive and pro-inflammatory peptide that contributes to endothelial dysfunction, fibrosis, and oxidative stress. In contrast, ACE2 serves as a counter-regulatory enzyme that degrades angiotensin II into angiotensin-(1–7), which exerts vasodilatory, anti-inflammatory, and protective effects. Importantly, ACE2 also serves as the cellular entry receptor for SARS-CoV-2, facilitating viral infection of host cells, particularly in the lungs.^65^, ^66^, ^67^ Viral binding to ACE2 reduces its membrane expression, disrupting the protective renin-angiotensin-aldosterone system balance and potentially exacerbating inflammation. While ACE2 polymorphisms may influence susceptibility to SARS-CoV-2 infection, ACE1 variants – such as the Insertion/Deletion (I/D) polymorphism – may affect disease severity by modulating angiotensin II-mediated inflammatory responses.^68^^,^^69^ Clarifying the distinct mechanisms of these enzymes is essential to understanding their respective contributions to COVID-19 risk and outcomes.

Almeida et al.^70^ investigated the impact of the ACE1 Insertion/Deletion (I/D) polymorphism on COVID-19 susceptibility and severity in a Brazilian cohort. The study included 70 severe cases (requiring high-flow oxygen, mechanical ventilation, or vasoactive support) and 355 mild cases. A significant association was found between the I/D polymorphism and both disease incidence and severity in male participants. Similar findings were reported in Egyptian children and adolescents, where the ACE1 D/D genotype and deletion allele were more frequent in COVID-19 patients than in controls (55 % vs. 28 %; OR = 2.4; 95 % CI 1.46 to 3.95).^71^ Additionally, the D/D genotype was identified as an independent risk factor for severe disease (adjusted OR = 2.6; 95 % CI 1.6 to 9.7; *p* < 0.001).^71^

Alaa et al.^72^ also reported that, among Egyptian patients, the GG genotype and wild-type allele of ACE2 rs908004, along with the mutant allele of ACE1 rs4343, were more prevalent in individuals with severe disease. Similar associations were observed in Iranian cohorts, where the D/D genotype correlated with an increased risk of severe COVID-19.^73^

Consistently, an Iranian study found that the ACE1 D/D genotype was associated with disease severity (OR = 2.00; 95 % CI 1.14 to 3.49),^74^ suggesting its utility as a predictive marker for severe COVID-19, particularly in individuals without known risk factors. Additional studies from Northern Cyprus^75^ and Mexico^76^ further supported the link between ACE1 D/D polymorphisms and severe disease outcomes.

Rezaei et al.,^77^ in a study of 120 hospitalized patients in Tehran, found that the ACE1 D allele was significantly associated with severe COVID-19 (OR = 6.76; *p* = 0.01), although no effect on mortality was observed. A meta-analysis including eight studies (1362 COVID-19 cases and 4312 controls) confirmed the association between the D allele and increased susceptibility to severe disease.^78^ Another meta-analysis showed that the ACE1 D/D genotype increased the risk of severe COVID-19 by 1.7-fold in Asian populations (95 % CI 1.11 to 2.76), while no significant association was observed in Western populations (OR = 1.36; 95 % CI 0.87 to 2.12; *p* = 0.17).^79^

In a cross-sectional Iranian study,^80^ the ACE2 rs2106809 G/G genotype was significantly more common in patients with severe disease compared to those with milder forms (44.4 % vs. 17.5 %; OR = 4.1; 95 % CI 1.8 to 9.5; *p* = 0.0007). Patients with this genotype were also more likely to require mechanical ventilation (*p* = 0.02), indicating its association with adverse outcomes.

Martínez-Gómez et al.^81^ identified the T-allele of ACE2 rs2285666 as a significant risk factor for severe and critical COVID-19, especially in men, regardless of age or comorbidities. Sabater Molina et al.^82^ confirmed these findings through multivariate analysis, emphasizing the critical role of this variant in determining disease severity. Further studies associated the CT + TT genotype of rs2285666 with poor clinical progression and post-COVID complications.^83^ Meta-analyses have supported these associations, showing that carriers of the ACE1 rs4646994 D/D genotype and the ACE2 rs2285666 G/G genotype are at increased risk for severe disease.^39^^,^^84^ Specifically, the rs4646994 deletion allele was associated with more severe manifestations (OR = 1.45; 95 % CI 1.26 to 1.66).^85^

Udomsinprasert et al.^50^ also reported an association between the ACE2 rs2285666 T-allele and a higher risk of developing long COVID. A descriptive longitudinal study investigating post-acute COVID-19 sequelae found that the A allele of ACE2 rs2106806 and the T-allele of rs6629110 were significantly associated with increased susceptibility to long COVID (OR = 4.21; 95 % CI 2.52 to 8.85; *p* < 0.001 and OR = 3.75; 95 % CI 1.78 to 6.10; *p* = 0.002, respectively).^86^

In summary, genetic studies and meta-analyses consistently underscore the pivotal role of ACE1 and ACE2 gene variants in influencing susceptibility to and severity of COVID-19. The ACE1 D/D genotype, in particular, is associated with a significantly increased risk of severe disease. Moreover, ACE2 polymorphisms such as rs2285666 and rs2106809 have emerged as potential predictors of both severe disease and long COVID, reinforcing the importance of these genetic markers in understanding COVID-19 outcomes and guiding personalized approaches to risk assessment and clinical management.

## Genes associated with COVID-19 protection

### OAS1

The OAS1 gene, particularly its splice-site single nucleotide polymorphism responsible for producing the p46 isoform, has emerged as a key factor in modulating COVID-19 severity.^87^ When activated, OAS1 binds to the double-stranded RNA structures of SARS-CoV-2, triggering viral RNA degradation and inhibiting replication. This antiviral mechanism is strongly associated with the G allele of the rs10774671 polymorphism.^88^

Evidence indicates that the rs10774671-G allele confers protection against severe COVID-19. Specifically, this allele is associated with a substantially reduced risk of severe disease (OR = 0.35; 95 % CI 0.15 to 0.83).^87^ Elevated expression of the OAS1 p46 isoform – favored by the G allele – is linked to favorable outcomes, including lower risks of death, mechanical ventilation, and hospitalization.^17^ This isoform possesses enhanced enzymatic activity, contributing to more effective viral clearance.^17^

The protective effect of the rs10774671-G allele has been observed across diverse populations. Among individuals of African descent, this allele is associated with a reduced risk of COVID-19 hospitalization (OR = 0.91; 95 % CI 0.85 to 0.98; *p* = 0.03),^88^ indicating a modest protective benefit. In contrast, the effect is more pronounced in European populations, where a stronger association has been observed between this allele and reduced risk of severe outcomes. This variation in effect size suggests that population-specific factors may influence the allele’s protective capacity.

In pediatric populations, children homozygous for the GG genotype have a significantly lower likelihood of developing severe disease compared to carriers of the A allele (OR = 5.71; 95 % CI 1.41 to 23.10),^89^ indicating that the A allele may confer greater vulnerability. Supporting this, Wickenhagen et al.^90^ demonstrated that the rs10774671 polymorphism influences the production of a more enzymatically active form of the OAS1 protein, thereby enhancing antiviral defense mechanisms.

These findings highlight the relevance of OAS1 gene variants as potential predictors of COVID-19 severity and reinforce the importance of further investigation into their role in viral pathogenesis. A deeper understanding of how OAS1 polymorphisms affect disease outcomes may contribute to the development of personalized therapeutic strategies and improved risk stratification in clinical settings.

### TMPRSS2

Research on the TMPRSS2 gene has revealed complex and sometimes conflicting associations with COVID-19 severity. While certain polymorphisms have been linked to increased disease severity, other variants appear to confer a protective effect.^91^^,^^92^

For example, the T-allele of the rs12329760 polymorphism has been associated with a reduced risk of severe COVID-19. A study conducted in the United Kingdom found that carriers of this allele had a 13 % lower risk of developing severe disease compared to non-carriers (OR = 0.87; 95 % CI 0.79 to 0.97).^91^ Similarly, a meta-analysis confirmed the protective association, reporting an OR of 0.77 (95 % CI 0.66 to 0.91) for the T-allele in preventing severe outcomes.^93^ Additional studies conducted in Egypt and East Asia supported these findings, demonstrating that the T-allele was less frequent among patients with severe disease compared to those with milder forms.^94^^,^^95^

In summary, the role of TMPRSS2 gene variants in COVID-19 severity appears multifaceted. While some polymorphisms are linked to increased vulnerability, others – such as the rs12329760 T-allele – may offer protection. These divergent findings underscore the complex interplay between genetic variation and clinical outcomes in COVID-19, highlighting the need for continued research to elucidate these associations and their potential implications for personalized treatment strategies.

### MUC5B

The MUC5B gene encodes a major mucin that contributes to airway mucus composition, playing a crucial role in maintaining respiratory health.^96^^,^^97^ The rs35705950-T polymorphism in MUC5B, a gain-of-function variant, has been investigated for its impact on COVID-19 severity.^98^^,^^99^

Several studies have suggested that the rs35705950-T variant is associated with a reduced risk of severe COVID-19. For example, data from the Million Veteran Program – a large and genetically diverse biobank – indicated that the T-allele conferred a protective effect against severe forms of the disease in European populations (OR = 0.82; 95 % CI 0.72 to 0.93).^100^ Similarly, a case-control study conducted in The Netherlands involving 108 COVID-19 patients and 611 healthy controls found that the T-allele was significantly less frequent among individuals with severe disease (OR = 0.75; 95 % CI 0.67 to 0.85),^101^ further supporting its potential protective role.

The role of MUC5B in mitigating severe COVID-19 is an emerging area of interest. Recent findings consistently associate the rs35705950-T polymorphism with reduced disease severity. This protective effect is hypothesized to result from enhanced mucociliary clearance and improved host defense mechanisms, although this has not yet been experimentally validated. One proposed mechanism suggests that increased MUC5B expression enhances the respiratory tract’s ability to clear pathogens, thereby reducing the viral load and severity of infections such as SARS-CoV-2.^102^^,^^103^ Elevated MUC5B levels may improve the efficiency of mucociliary clearance, a critical component of the innate immune defense against respiratory viruses.

Although the exact biological mechanisms remain to be fully elucidated, the observed association between MUC5B polymorphisms and decreased COVID-19 severity underscores the gene’s potential role in respiratory defense. Further research is warranted to validate these hypotheses and to assess the viability of MUC5B as a candidate gene for therapeutic targeting and risk stratification in COVID-19.

### TYK2

The TYK2 gene, previously discussed for its association with increased COVID-19 severity, also harbors polymorphisms that may confer a protective effect. Importantly, research by Zabihi Rizi et al.^35^ identified specific alleles within TYK2 associated with reduced disease severity. The rs2304256 allele and the rs12720270 A allele were both significantly linked to a lower risk of severe COVID-19, with ORs of 0.25 (95 % CI 0.16 to 0.38) and 0.17 (95 % CI 0.11 to 0.26), respectively.

These findings suggest that while certain TYK2 variants are associated with increased susceptibility to severe COVID-19, others may exert a protective effect. This duality underscores the complex role of TYK2 in immune regulation and highlights the importance of considering variant-specific effects when evaluating the gene’s impact on COVID-19 outcomes.

### GNB3

The G-protein subunit Beta 3 (GNB3) gene has been identified as a potential modulator of immune responses in various conditions, including viral infections.^104^ In a study by Möhlendick et al.,^105^ the impact of the rs5443 single nucleotide polymorphism in GNB3 was investigated, suggesting that this genetic variation may enhance T-cell activity, thereby contributing to improved immune responses and reduced COVID-19-related mortality.

It has been hypothesized that the rs5443 polymorphism affects G-protein signaling pathways, which play a critical role in the regulation of immune cell activity, including T-cell function. Enhanced G-protein signaling may lead to increased T-cell activation and a more effective antiviral response, potentially improving the host’s ability to control SARS-CoV-2 infection. However, these findings remain preliminary, and the precise molecular mechanisms through which GNB3 influences T-cell function and COVID-19 outcomes are not yet fully understood.

Despite these promising insights, further research is needed to elucidate the mechanistic pathways linking GNB3 variants to immune modulation and to determine their potential role in shaping clinical outcomes in COVID-19.

### Final considerations

The interplay between genetic factors and COVID-19 has revealed a complex landscape of both risk and protection. The main genes and polymorphisms associated with the clinical presentations of acute COVID-19 and long COVID discussed in this review are summarized in Table 1.

**Table 1:** Genes and polymorphisms associated with clinical presentations of acute and long COVID.

	Genes	Polymorphisms	Associated Outcomes
**Increased Risk**	TMPRSS2	rs2070788 (GG genotype)	**↑** Risk of death
	OAS1	rs6489867, rs10735079, rs4767027	**↑** Risk of SARS-CoV-2 infection
	TYK2	rs74956615, rs2304255 (T allele)	**↑** Risk of severe COVID-19
	MBL2	AB genotype, BB genotype	**↑** Risk of severe COVID-19 and ICU
	FOXP4	rs1886814, rs2894439	**↑** Risk of severe COVID-19
	IFNAR2	rs3153, rs1051393, rs2236757, rs2834158	**↑** Risk of death
	ACE1	D/D genotype, rs4646994	**↑** Risk of severe COVID-19
	ACE2	rs2106809 (G/G), rs908004, rs2285666 (T allele)	**↑** Risk of severe COVID-19
**Protective Effect**	TMPRSS2	rs12329760	**↓** Risk of severe COVID-19
	MUC5B	rs35705950-T	**↓** Risk of severe COVID-19 and hospitalization
	TYK2	rs2304256, rs12720270 (A allele)	**↓** Risk of severe COVID-19
	OAS1	rs10774671 (G allele), GG genotype	**↓** Risk of severe COVID-19
	GNB3	rs5443	**↓** Risk of death

ICU: Intensive Care Unit.

Over the past few years, research has illuminated how various genetic variants can influence susceptibility to infection, severity of disease, and even long-term outcomes. For instance, some polymorphisms affect immune responses, while others impact viral entry efficiency or modulate inflammatory pathways. Understanding these genetic mechanisms at the molecular level is essential for exploring their potential clinical and biological implications. Nonetheless, such findings should be interpreted with caution, as many reported associations may be correlative rather than causative and are often influenced by a range of non-genetic factors.

This expanding body of evidence not only aids in identifying individuals at increased risk but also paves the way for personalized medicine strategies in the management and treatment of COVID-19. For example, if specific genetic variants are shown to influence immune regulation, therapies targeting these pathways – such as cytokine modulators – could be considered for patients with high-risk genotypes. Likewise, insights into gene mutations involved in viral entry or replication may inform antiviral strategies, including drug repurposing or the development of new therapeutic agents tailored to individual genetic profiles.

The investigation of genetic determinants related to COVID-19 severity and protection remains a dynamic and evolving field. Substantial progress has been made in identifying how genetic variation can influence outcomes, but it is essential to recognize that genetics is only one component of a multifactorial framework. Age, comorbidities, ethnicity, and environmental factors also play critical roles. The interaction between genetic and non-genetic factors adds complexity to the understanding of risk assessment.

**Fig. 1:**
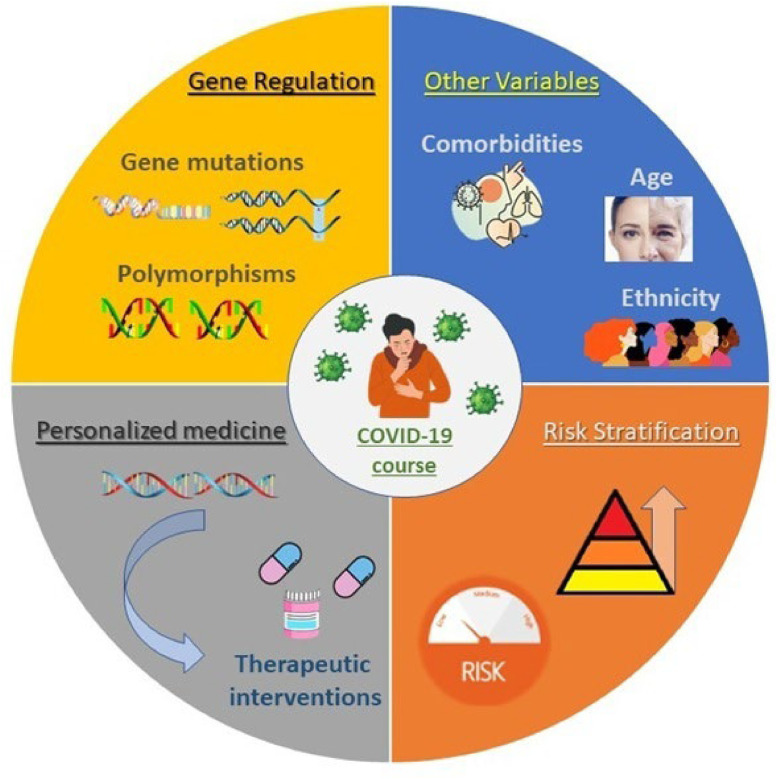
The intersection of genetic factors in COVID-19. Graphic illustration of the intersection between genetic regulation and risk factors, and the potential of personalized medicine to optimize treatment strategies and improve outcomes in the COVID-19 population.

By elucidating the genetic mechanisms underlying COVID-19 susceptibility and progression, researchers aim to develop more accurate tools for risk stratification and more precise therapeutic interventions (Fig. 1). This could ultimately lead to more proactive and effective disease management strategies that benefit patients on a global scale.

Continued research in this field holds great promise for advancing personalized medicine, guiding preventive measures, and enabling the development of novel therapeutics tailored to individual genetic backgrounds. It may also identify genetic markers predictive of treatment or vaccine responsiveness, allowing for more targeted and efficient interventions. As our understanding deepens, the integration of genetic data into clinical practice has the potential to significantly improve COVID-19 outcomes through individualized approaches.

## Conclusions

Genetic variations and interindividual differences may contribute to varying susceptibilities to SARS-CoV-2 infection and to differences in COVID-19 severity. However, these associations are likely shaped by a complex interplay of environmental exposures, lifestyle factors, and additional biological mechanisms. Genes involved in viral entry and immune regulation highlight the multifactorial nature of genetic contributions to disease outcomes.

Moreover, while substantial progress has been made in identifying variants associated with acute COVID-19, research on the genetic determinants of long COVID – particularly regarding recovery trajectories and persistent symptoms – remains limited. Further studies are needed to elucidate these associations and to better characterize the genetic architecture underlying both acute and long-term COVID-19 outcomes.

## Informed consent statement

Not applicable. This study is a narrative review and did not involve human participants or the collection of individual data.

## Institutional review board statement

Not applicable. This study is a narrative review and did not involve human participants or require ethical approval.

## Funding

The Article Processing Charge for the publication of this research was funded by the Coordenação de Aperfeiçoamento de Pessoal de Nível Superior (CAPES), Brasília, Brazil; the 10.13039/501100003593National Council for Scientific and Technological Development (CNPq), Brasília, Brazil (Grant No. 444823/2023-9); and the Research Incentive Fund of Hospital de Clínicas de Porto Alegre (FIPE-HCPA), Porto Alegre, Brazil.

## CRediT authorship contribution statement

**Thais Beuren:** Conceptualization, Data curation, Validation, Visualization, Writing – original draft. **Filipe Ferrari:** Conceptualization, Data curation, Validation, Visualization, Writing – original draft. **Leandro Tolfo Franzoni:** Data curation, Validation, Visualization, Writing – original draft. **Cássia da Luz Goulart:** Data curation, Validation, Visualization, Writing – original draft. **Fernando Val:** Data curation, Validation, Visualization, Writing – original draft, Writing – review & editing. **Gerson Cipriano:** Data curation, Validation, Visualization, Writing – original draft. **Ricardo Stein:** Conceptualization, Data curation, Validation, Visualization, Writing – original draft, Writing – review & editing.

## Declaration of competing interest

The authors declare no potential conflicts of interest concerning the research, authorship, and/or publication of this study. Thais Beuren receives financial support from the Coordination for the Improvement of Higher Education (CAPES), Brasília, Brazil. Filipe Ferrari, Leandro Tolfo Franzoni, and Cássia da Luz Goulart receive financial support from the National Council for Scientific and Technological Development (CNPq), Brasília, Brazil. Ricardo Stein, Fernando Val, and Gerson Cipriano Jr. are Established Investigators of the CNPq, Brasília, Brazil. Gerson Cipriano Jr. also receives financial support from the Research Support Foundation of the Federal District (FAPDF), Brasília, Brazil.
